# Complementary Therapies for Significant Dysfunction from Tinnitus: Treatment Review and Potential for Integrative Medicine

**DOI:** 10.1155/2015/931418

**Published:** 2015-09-20

**Authors:** Ruth Q. Wolever, Rebecca Price, A. Garrett Hazelton, Natalia O. Dmitrieva, Elizabeth M. Bechard, Janet K. Shaffer, Debara L. Tucci

**Affiliations:** ^1^Department of Physical Medicine & Rehabilitation, Osher Center for Integrative Medicine, Vanderbilt University Schools of Medicine and Nursing, 3401 West End, Suite 380, Nashville, TN 37203, USA; ^2^Duke Integrative Medicine and Duke Department of Psychiatry and Behavioral Sciences, Duke University School of Medicine, DUMC 102904, Durham, NC 27710, USA; ^3^Department of Speech Pathology & Audiology, Duke University Health Systems, DUMC 3887, Durham, NC 27710, USA; ^4^Department of Psychiatry and Behavioral Medicine, East Carolina University, 600 Moye Boulevard, Greenville, NC 27834, USA; ^5^Department of Psychological Sciences, Northern Arizona University, P.O. Box 15106, Flagstaff, AZ 86011, USA; ^6^Duke Integrative Medicine, DUMC 102904, Durham, NC 27710, USA; ^7^Division of Head and Neck Surgery & Communication Sciences, Duke University School of Medicine, DUMC 3085, Durham, NC 27710, USA

## Abstract

Tinnitus is a prevalent and costly chronic condition; no universally effective treatment exists. Only 20% of patients who report tinnitus actually seek treatment, and when treated, most patients commonly receive sound-based and educational (SBE) therapy. Additional treatment options are necessary, however, for nonauditory aspects of tinnitus (e.g., anxiety, depression, and significant interference with daily life) and when SBE therapy is inefficacious or inappropriate. This paper provides a comprehensive review of (1) conventional tinnitus treatments and (2) promising complementary therapies that have demonstrated some benefit for severe dysfunction from tinnitus. While there has been no systematic study of the benefits of an Integrative Medicine approach for severe tinnitus, the current paper reviews emerging evidence suggesting that synergistic combinations of complementary therapies provided within a whole-person framework may augment SBE therapy and empower patients to exert control over their tinnitus symptoms without the use of medications, expensive devices, or extended programs.

## 1. Introduction

Tinnitus is a prevalent problem for which there is no universally effective treatment. The best available estimates indicate that 10–15% of adults report tinnitus symptoms across Europe, the United States (US), Japan, and several African countries [1, 2]. Tinnitus is also quite costly; the US Department of Veterans Affairs (VA) reported that 289,159 veterans received a disability award for their tinnitus in 2004, which amounted to an annual compensation of over $345.5 million [3]. An estimated 20% of those who report tinnitus suffer from it and subsequently seek treatment [1]. After treatable causes have been managed, the standard care for tinnitus treatment in most US otolaryngology and audiology clinical practices consists of sound-based and educational (SBE) therapy, with additional interventions to reduce distress provided as needed [2].

The first aim of the current paper is to offer researchers and treatment providers a review of the conventional treatments for dysfunction from tinnitus. While SBE treatments provide a starting point for tinnitus treatment, SBE therapy is not appropriate or effective for patients with poor hearing or a lack of habituation to sound and can be difficult for patients who experience problems with sound tolerance. Moreover, a tinnitus treatment is sorely needed that also addresses the significant nonauditory aspects of tinnitus (e.g., anxious and depressive symptoms, sleep problems, and significant interference with daily life [4]). Thus, the second aim of the current paper is to review evidence-based complementary therapies that show promise in a population of patients with tinnitus. Availability of efficacious complementary strategies would allow providers to augment currently used therapies in order to empower patients to exert control over their tinnitus symptoms without the use of medications, expensive devices, such as the Neuromonics device, or extended programs such as Tinnitus Retraining Therapy. We argue that promising complementary approaches may augment conventional treatments for all tinnitus patients and may be particularly useful for patients who are not candidates for SBE therapy, for those who respond poorly to SBE therapy, and for those who exhibit significant nonauditory symptoms of tinnitus (e.g., depressive symptoms).

## 2. Conventional Treatments

### 2.1. Sound-Based and Educational (SBE) Therapy

Used in the majority of tinnitus treatment programs in the US, SBE therapy consists of education about the mechanism of tinnitus to provide a rationale for sound-based therapy and is modeled on the neurophysiological theory of habituation to the tinnitus. One widely used SBE approach is that of the US VA called Progressive Tinnitus Management (PTM) [5]. SBE treatment incorporates the use of educational counseling and stress management, along with the integration of sound therapy to better manage the impact of tinnitus. The goal of this approach is to provide the patient with the education, resources, and formalized counseling to be able to self-manage their tinnitus [6]. The therapy can stand alone or provide a basis to combine with other treatments [5].

#### 2.1.1. SBE Counseling and Stress Management

Education provided in SBE therapy can be provided in individual or in group settings. It typically entails a review of the neurophysiological model of tinnitus, as well as dietary and stress triggers that can exacerbate symptoms. For example, in the VA's PTM [7], participants are given self-management workbooks entitled* How to Manage Your Tinnitus: A Step-by-Step Workbook* as part of Level 2 Audiologic Evaluation. Patients have the opportunity to think through those triggers that individually affect them and to structure plans to modify their exposure to such triggers by creating achievable and relevant goals. In addition, the “Sound Plan Worksheet” [5, Appendix  A] from these workbooks helps the patient determine what scenarios were “most bothersome” and formulate an action plan for each scenario with a focus on utilizing sound enrichment to reduce tinnitus disturbance [6].

#### 2.1.2. SBE Sound Enrichment

Use and type of sound are described to patients to help determine what type of sound will be best at tinnitus reduction in different scenarios.* Soothing sound* is described as a sound where the patient experiences a sense of relief with increased relaxation when the sound is on [8]. Examples include ocean-like sounds or slow tempo music. The use of soothing sound is considered a therapeutic intervention to reduce feelings of distress associated with tinnitus.* Background sound* is meant to reduce the contrast effect of tinnitus. This may simply be use of white noise or background television noise where the focus is not on the sound itself but the presence of a sound, which reduces the contrast of the patient's tinnitus in a quiet environment [8].* Interesting sound* uses auditory input in combination with context, to not only provide a sound source, but also actively refocus the patient's attention away from the tinnitus by presenting interesting sounds. Examples of interesting sound might include watching television or listening to a podcast [8]. Environmental sound, music, and speech can be used for each of the three categories of sound described above. During SBE therapy, participants are encouraged to explore these three uses of sound to determine which sound may provide the most relief for them in their most bothersome tinnitus scenarios [5]. Using tracking worksheets, such as the Sound Plan Worksheet described above, participants monitor use of specific sound enrichment attempts for each type of sound and rank how helpful each is in managing their tinnitus for at least one week, using a 5-point Likert scale that ranges from “not at all” (0) to “extremely effective” (4). These efficacy ratings for sound enrichment at home are then reviewed with the audiologist, and patients are recounseled if necessary on tinnitus education and given additional support to maximize their use of sound therapy. For instance, if patients indicated that they only experienced a “mild relief” using background sound when falling asleep at night, they would be counseled on exploring another category of sound, such as soothing sound, which may be more beneficial in that particular tinnitus scenario. Similarly, if a patient found that they did not get enough relief when using a particular method or device, additional options are described, including a docking station for smartphone/iPod/MP3 player, a pillow speaker with auxiliary connection, and earbuds.

## 3. Complementary Therapies

Nonconventional approaches for tinnitus have increased in prevalence and acceptance among both patients and practitioners. Nonetheless, mainstream medical journals report that there is no evidence that such methods have reduced tinnitus volume or associated distress [2]. Although research support of nonconventional therapies for tinnitus has been limited, there is increasing justification for including them with SBE therapy. In particular, there is growing evidence for the following approaches: (1) psychological and behavioral therapies, such as cognitive-behavioral therapy (CBT) [9–11]; (2) mind-body therapies, such as biofeedback and meditation, which target autonomic balance and can be effectively coupled with CBT to independently address psychological and physiological variables [12–14]; and (3) acupuncture [15, 16].

Many of these approaches have been shown to benefit some tinnitus sufferers, but none have shown efficacy in the majority of tinnitus patients, leaving providers in the position of suggesting therapies that have the greatest likelihood of success for individual patients. Complementary treatments may be particularly well suited for treating the dysfunction associated with tinnitus, as they specifically target aspects of tinnitus that are often overlooked in conventional medicine. Hence, the following review provides clinicians with a summary of the evidence base for complementary therapies that show promise in a population of patients with severe dysfunction from tinnitus and, in particular, for nonauditory aspects of tinnitus symptoms. In addition, complementary strategies may be more broadly applied to people from various cultural backgrounds. Hence, complementary strategies may be useful for patients who are not candidates for SBE therapy, for those who respond poorly to SBE therapy, and for those who exhibit significant nonauditory symptoms of tinnitus.

### 3.1. Cognitive-Behavioral Therapies

Despite some equivocal findings, the majority of evidence considers CBT an effective treatment of the distress associated with tinnitus [17] and may include training in activity planning, relaxation exercises, and cognitive restructuring [9, 10]. Results of CBT trials have been demonstrated to persist in the long term, with several studies showing benefit at 12 months and beyond active treatment [18–20]. A recent Cochrane review examined 6 randomized clinical trials that investigated the use of CBT for tinnitus and concluded that while there was no demonstrable effect on subjective tinnitus loudness or the depression associated with tinnitus, treatment did reduce the global severity of tinnitus and improve quality of life [17]. Two recent systematic reviews have used comprehensive methodologies and had somewhat differing outcomes. The first systematic review searched 6 psychological and medical data bases from 1970 to June 2012 and also included an extensive grey literature search of the same time period. These researchers found the evidence for CBT as an intervention to improve tinnitus-specific quality of life relative to inactive controls to be of low strength [17]. In the second systematic review, 31 experimental studies, randomized trials, and reviews of treatment approaches were identified from 13 data bases and registries in psychological and medical peer-reviewed literature between 1966 and October 2012. Findings from this group of studies confirmed that CBT is the most evidence-based treatment option thus far [11]. Despite diverse treatment elements and varied outcome assessments, the reviewers strongly concluded that the evidence on a common ground of therapeutic elements was robust enough to guide clinical practice.

Psychotherapists well trained in CBT help patients recognize and explore the relationship of their thoughts with other aspects of their experience, including emotions, physical symptoms, and behaviors. This approach is relevant to tinnitus, as the psychological distress that follows the development of tinnitus has been well documented, and recent research has proposed that distress is a possible mechanism in the development and worsening of tinnitus [21]. In CBT for tinnitus, patients often discuss their history with tinnitus symptoms, and clinicians assess their knowledge of tinnitus with a focus on the biopsychosocial effects of tinnitus and the individual's storyline of the development and experience of living with the disorder. During patient recall, their “tinnitus narrative” is explored with prompts such as the following. (1) How did the tinnitus begin/develop? (2) What factors might have prevented development of the tinnitus? (3) Do you blame someone or something for the tinnitus? (4) What are the effects of tinnitus? (5) What are the negative aspects of having tinnitus? (6) What are the benefits related to having tinnitus? Next, patients begin the initial steps of cognitive restructuring, that is, exploration of patient beliefs about tinnitus and common negative automatic thoughts related to the condition. In some cases, corrective thinking is needed when the patient's information is inaccurate; for example, “Tinnitus is making my hearing worse”; “Tinnitus is causing some other damage such as dizziness”; “Tinnitus means I am going crazy.” In other cases, simple awareness of thoughts is encouraged, as the patient and clinician process potential differences or discrepancies between the tinnitus and the patient's story about tinnitus. CBT typically also includes structured, self-monitoring strategies [12] with the goal of patients increasing their awareness of the nature and effects of their tinnitus and in particular how these factors relate to their own cognitions, emotions, and behaviors. Regarding self-monitoring, clinicians must also be careful to educate more ruminative patients on the possibility of excessive monitoring, or a tendency to continuously self-monitor intensity or quality of sound at the cost of distraction from other important activities or experiences [22]. In this case, clinicians might note how the mind may naturally increase its sensitivity to anything that is worrisome (such as tinnitus) and therefore if excessive monitoring became problematic, patients are encouraged to set goals around issues other than self-monitoring and to encourage friends and family (e.g., spouse) to not ask how the tinnitus is.

CBT sessions can also be used to introduce patients to the behavioral principle of exposure. Patients are informed that if a fear response to sounds occurs, exposure to environmental sounds may be helpful in reducing emotional reactivity. Clinicians and patients then work together to design an exposure and response prevention plan as needed [23]. Patients with sleep hygiene challenges can be educated on healthy sleep habits and realistic goals to apply sleep hygiene practices. Finally, CBT can be used to teach the patients relaxation training (e.g., progressive muscle relaxation, diaphragmatic breathing, and/or guided imagery), with the goal of reducing the negative effects of tinnitus rather than reducing the tinnitus sound itself.

### 3.2. Mindfulness-Meditation

Processes that target physiological activity can also help to address the hyperreactivity that hinders the habituation process [13]. Emerging evidence suggests that mindfulness-meditation may be one such process. Across the board, the past decade has produced a burgeoning evidence base on the benefits of mindfulness-meditation based approaches to multiple other conditions [24–29]. Mindfulness-meditation trains participants in attention and teaches them to observe sensations, thoughts, feelings, and behavioral urges from an objective nonjudgmental stance [24, 30, 31]. This attentional training further teaches participants to neutrally observe and separate the actual event (e.g., a tinnitus sound) from psychological or cognitive reactions to this event (e.g., an interpretation of sound or distress about it). The most researched form of training in mindfulness is through a structured program of Mindfulness-Based Relaxation Training (MBSR) [30, 32]. MBSR is a method of using meditation and yoga to cultivate present moment awareness and reduce stress. It is based on the ancient practice of mindfulness, which is about waking up and being fully present in the moment. From this stance, patients are able to learn about how their minds work through directly observing their own sensations, thoughts, emotions, and urges to behave.

The evidence for mindfulness-meditation as an adjunctive treatment comes from two sources. First, there is particularly strong evidence from RCTs for the effectiveness of mindfulness-based approaches to chronic pain [33–38], which has striking similarities to chronic tinnitus from an adaptation perspective [39]. Specifically, both have a physiological sensory component that is particularly distressing when their negative affective reactions are coupled with negative cognitive appraisals (e.g., “This will never end. I'll lose my mind”). The second source of evidence comes from three published studies. The first study integrated mindfulness-meditation as a concentration exercise in one of the CBT treatment sessions; however mindfulness did not play a salient role in this work, making it difficult to tease apart the contribution of this approach [9]. Two more recent studies, however, focused more specifically on the potential benefits of mindfulness training for tinnitus patients. Both trials were small (*n* = 8 and 25) and had methodological limitations: one had no control group [14], while the other consecutively allocated participants to conditions rather than randomizing them [40]. Nonetheless, both pilot trials showed that mindfulness-meditation resulted in changes in the expected direction on a number of outcomes, including subjective tinnitus severity. Hence, both studies provide useful preliminary data on potential mechanisms that warrant inclusion of mindfulness as a complementary therapy for tinnitus.

### 3.3. Acupuncture

Although methodological difficulties in tinnitus-related acupuncture literature remain, recent systematic reviews and one meta-analysis suggest that acupuncture may offer subjective benefit to some tinnitus patients. Evidence exists that acupuncture is beneficial in some conditions, such as postoperative dental pain [41], osteoarthritis of the knee [42], tension-headaches [43], and postoperative as well as chemotherapy-related nausea and vomiting [41]. It is of possible but unproven benefit for conditions such as migraine, low back pain, and temporomandibular joint dysfunction [41]. Findings on the impact of acupuncture in tinnitus are slightly more equivocal. An older yet methodologically rigorous review of six RCTs on the use of acupuncture for tinnitus treatment determined that acupuncture had not been demonstrated to be efficacious [44]. The two RCTs that reported a benefit were unblinded studies. A more recent study however did report a significant difference in otoacoustic emission amplitude measured in tinnitus patients before and after temporoparietal acupuncture (targeting the vestibulocochlear area) as opposed to acupuncture in a sham area (not a recognized acupuncture point) [45]. Two more recent systematic reviews also show equivocal findings. In the first, 9 RCTs were identified from 14 data bases through July 2012 that used acupuncture as the sole treatment for tinnitus. The investigators concluded that the methodological quality of the RCTs was mostly poor and not sufficient to draw conclusions of the effectiveness of acupuncture for tinnitus [16]. Similarly, in the second systematic review, RCTs that evaluated acupuncture treatment against no treatment, sham treatment, medications, or other medical therapy were identified from five electronic data bases in English and Chinese between 1966 and 2013. In this review, the authors found that most of the Chinese studies had methodological flaws, including risk bias, but reported positive results and may use more appropriate acupoints and processes. On the other hand, most of the English studies reported negative results and used distinct acupoints compared to those utilized in the Chinese studies. An analysis of all data together suggested that acupuncture provided some advantages over conventional therapies for tinnitus in terms of subjective benefit but agreed that no definitive conclusions could be made [15].

Licensed acupuncturists trained in Traditional Chinese Medicine (TCM) use an individually tailored, whole-person approach guided by TCM theory to determine the specific acupuncture points needed for each participant. Individuals are examined with a variety of diagnostic techniques, including observation of movement and complexion, careful listening to their voice quality and description of their overall health, and asking about health history and additional complaints, while noting participant emotions and opinions about the onset and their current subjective experience of tinnitus sounds and sensations. Selection of acupuncture points is informed by these diagnostic indicators, when considered along with proper differentiation of the subjects' constitutional type per TCM theory. In addition to differentiating constitutional type, acupuncture point selection is also influenced by the origin, rate of onset, and different mitigating or exacerbating factors specific to the experience of tinnitus. Furthermore, acupoint selection and the style of needle insertion are informed by the patient's body type, age, lifestyle, diet, and environment. The acupuncturist uses all of these clues to make adjustments to ease and alter general health issues, while simultaneously addressing tinnitus.

Essential TCM principles relevant to understanding commonly used acupoints include the following: (1) the distinction in Full type versus Empty type conditions: Full conditions respond to reducing or draining techniques while Empty conditions reflect deficiencies associated with fatigue, age, or overworking; (2) organ systems involved in the Fullness or Emptiness. Per best-practices in acupuncture, selection of acupoints is done to balance historic and constitutional factors as well as those points indicated as a function of the tinnitus per traditional or modern texts for their indicated effects and proximity to the ears. Although there are no standardized points used for every patient, several common constitutional and “tinnitus” points are used in most patients when appropriate. These include the following:

- SI-19, ting gong: indicated for tinnitus and located in front of the ear;
- SJ-17, yi feng: indicated for tinnitus, usually of Full type, and located behind the ear lobe;
- KID-3, tai xi: indicated to nourish yin, useful in Empty conditions;
- SP-6, san yin jiao: indicated to calm nervous system, useful in “nourishing blood” in Empty conditions in which this is a salient feature.

Based on current evidence, it appears that using acupuncture as an alternative treatment* instead* of conventional treatment is not warranted. Using acupuncture as a* complementary* therapy however may be worthwhile. It is not uncommon that allopathic medicine patients perceive medical treatment as something that they have “done to them” and thus expect to take a more passive role as is more typical in conventional medicine (versus CAM) [46]. In shifting patients to a more patient-centric and active role wherein they can take responsibility for managing symptoms, it seems prudent to include treatment aligned with their expectations at the same time as introducing new perspectives. In addition, patients with limited understanding of psychologically based treatments often object to these treatments because of the misconception that providers are suggesting treatment because the symptom is imagined or “in my head.” Hence, acupuncture may be worthwhile to ease perceptual shift to an empowered patient model, while also increasing subject adherence and belief in potential treatment efficacy.

### 3.4. Synergistic Combinations of Therapies

Two recent reports provide support for the potential utility of combining complementary treatment modalities to reduce tinnitus symptomatology. First, using strong methodology, Philippot et al. published a randomized controlled trial evaluating a single group 2-hour session of psychoeducation followed 2.5 months later by six weekly sessions of either mindfulness or relaxation training. Psychological benefits obtained in the psychoeducation training were maintained in the mindfulness condition while they were eroded in the relaxation condition at 3-month follow-up [47]. Second, a new comprehensive literature review that examined tinnitus treatment studies from psychological and medical data bases over the last 3 decades suggested that tinnitus treatment be CBT-based, while moving to a more multidisciplinary approach [11].

There are a number of reasons that combinations of therapies make sense for patients with tinnitus. First, those who suffer from severe tinnitus represent a wide variety of individuals with highly distinct characteristics [48, 49]. No single treatment is likely to address all sources of tinnitus-related dysfunction. Second, there is an opportunity to synergize treatment components in a meaningful way. For example, by combining SBE therapy (usually performed by audiologists) and CBT (provided by psychotherapists), there is a chance to deepen patient's exploration and learning about individual factors that might improve or exacerbate tinnitus symptoms. The different disciplines (audiology and psychology) can also help patients address their observations from distinct perspectives. Monitoring that is first taught in SBE therapy, for example, could be later reviewed in CBT to enhance patient learning about the frequency, quality, and volume of tinnitus, as well as associations with thoughts, emotions, and behavior. As another example, CBT in combination with mindfulness-meditation could help patients deepen their personal motivation for pursuing treatment while also increasing awareness of the role of their thoughts and emotions in managing tinnitus, setting the stage for participants to become curious about the practice of mindfulness. Learning to take an observational stance through mindfulness is significantly aided by participant ability to note thoughts, as learned in CBT. The reverse is also true.

### 3.5. Considerations for Order of Therapies

In an ideal setting, providers work in an integrated framework, based on each provider's knowledge of the content and process of the other therapies. It may be most efficient to have patients begin treatment with SBE therapy. If the SBE therapy is not adequate to manage their needs, they may benefit from referral for CBT. During CBT, patients may be reminded of the SBE discussion and can explore with the therapist what factors might make tinnitus better or worse. In both SBE therapy and CBT, daily diaries can be used, with directions for indicating the frequency, quality, and volume of tinnitus, as well as space for indicating mood, anxiety, and coping behavior. Self-management of these issues may then be explored further in CBT, mindfulness-meditation, and other therapies.

## 4. Discussion

The use of an Integrative Medicine (IM) framework may allow for the synergy needed between conventional treatment and complementary therapies in patients with tinnitus. A further advantage is that IM therapies may provide a global approach to treatment that allows for selection of the most effective strategies for management of individual patients. It has long been observed that tinnitus sufferers vary in tinnitus characteristics and responses to various therapies, leading many to hypothesize that there are a number of tinnitus subtypes [48]. As there is not currently an accurate characterization of subtypes, treatments are offered on an exploratory basis; in current practice these are most commonly offered sequentially rather than in an integrative and coordinated fashion. Integration of therapies into a programmatic whole with subsequent selection of those most effective into an individualized program of tinnitus management may be the most effective approach. An even more effective IM approach could allow for streamlined treatment that individualizes tinnitus intervention based on symptoms and patient characteristics and that can be widely applied in general medical practice. More study is required to determine how to best build and then optimize such a patient treatment program. Subsequent studies must include randomized controlled trials which control for the potential placebo effects that have traditionally plagued trials of complementary and alternative approaches.

### 4.1. Theoretical Framework of IM

Integrative Medicine is often confused with Complementary and Alternative Medicine (CAM), but it is much broader. A recent large scale, international review defined IM as “CAM provided holistically and in conjunction with conventional medicine” [50]. Treatments in IM are also provided through a distinct framework. Discriminating features of the framework include individual-tailoring of holistic interventions (e.g., targeting multiple aspects of one's life simultaneously), the incorporation of mind-body techniques such as meditation, and delivery of all interventions through a patient-centered context wherein the patients' goals, interests, and values inform the treatment [51]. These key tenets of IM align well with what is known about those with severe tinnitus. First, since tinnitus patients demonstrate highly diverse characteristics [49], it is reasonable to assume that individually tailored holistic approaches would be of benefit. Second, those with severe tinnitus tend to demonstrate greater distress and psychological vulnerabilities [13, 20, 52], which would predictably be ameliorated by patient-centered care. The benefits of such care in providing symptom relief are well established [53–58]. Third, individuals with greater distress and psychological vulnerabilities have been consistently shown to benefit from both CBT and mind-body approaches [59–62]. Finally, it has been noted that monotherapies have been less effective for tinnitus than have combination therapies [47], particularly since tinnitus affects a broad spectrum of symptoms and individuals [63].

Previous trials have demonstrated effectiveness of synergistic IM approaches combining the complementary therapies noted in this paper that are likely to assist with dysfunction from tinnitus. For example, in a prospective observational trial, an IM treatment that combined education, mindfulness-meditation, acupuncture, massage, nutrition consultation, fitness training, and health coaching proved effective in reducing prospective risk of stroke and diabetes through small improvements in multiple risk parameters (e.g., anger, anxiety, depression, social support, exercise behavior, resting pulse, body mass index, waist circumference, and cholesterol) [64]. As another example, in a randomized controlled trial (RCT) that compared a synergistic IM intervention to usual care, an IM intervention was shown to be effective in reducing 10-year prospective risk of coronary heart disease [32]. Again, small improvements in multiple parameters lowered prospective risk in the IM intervention which combined education (on nutrition and physical activity), mindfulness-meditation (as well as other mind-body approaches), CBT-based skill-building (stress management, goal setting, assertiveness, and relapse prevention), and health coaching [32].

## 5. Conclusions

Most tinnitus patients enter the health care system through traditional medical providers, which may include primary care providers, otolaryngologists (ENT doctors), and audiologists. Collaborative care pathways should be established to increase options for tinnitus patients who may benefit from a variety of available therapies and to help to define which patients would benefit most from a holistic, IM approach. Further studies are needed to demonstrate efficacy of the IM approach to tinnitus and to allow for individualization and refinement of treatment programs.
